# Alkali metal salts of 4-hy­droxy­benzoic acid: a structural and educational study

**DOI:** 10.1107/S2053229621005465

**Published:** 2021-06-09

**Authors:** Brendan F. Abrahams, Christopher J. Commons, Timothy A. Hudson, Robin Sanchez Arlt, Keith F. White, Michael Chang, John J. Jackowski, Matthew Lee, Shang X. Lee, Harrison D. Liu, Bill M. Mei, Joshua E. Meng, Lincoln Poon, Xiaolin Xu, Zekai Yu

**Affiliations:** a School of Chemistry, University of Melbourne, Parkville, VIC 3010, Australia; b School of Molecular Science, La Trobe University, Wodonga, VIC 3690, Australia; c Scotch College, 1 Morrison Street, Hawthorn, VIC 3122, Australia

**Keywords:** crystal structure, short strong hydrogen bond, SSHB, hydrogen-bonded network, crystallographic education, hy­droxy­benzoic acid, bilayer, ionic network, crystal engineering

## Abstract

As part of an educational exercise designed to introduce school students to the technique of single-crystal X-ray diffraction and enhance their understanding of primary and secondary bonding, a group of nine secondary school students was given the opportunity to prepare new com­pounds and to solve and refine data collected on the crystalline materials they had prepared. Their investigation of the alkali metal salts of 4-hy­droxy­benzoic acid (H_2_hba) yielded nine new com­pounds and their structures are described in this article. Whilst the salts might be expected to have similar atomic arrangements, there are significant differences in their structures. Although H_2_hba is a relatively simple organic mol­ecule, it displays remarkable coordinative flexibility, forming ionic solids containing the uncharged mol­ecule, the monoanion Hhba^−^ or the dianion hba^2−^. A common feature of the structures is their layered arrangement: alternating hydro­philic layers made up of closely packed metal–oxygen polyhedra separated by the hydro­phobic com­ponent of the hy­droxy­benzoate linking units. Close packing of these units seems to be a dominant influence in determining the overall structure. The hy­droxy­benzoate units are usually both parallel and anti­parallel with their immediate neighbours, with packing that can be edge-to-face, face-to-face or a mixture of the two. Hydrogen bonding plays a key role in the structure of most com­pounds and a short strong hydrogen bond (SSHB) is observed in two of the networks. The com­pounds of 4-hy­droxy­benzoic acid, C_7_H_6_O_3_, described here are: poly[di-μ-aqua-μ-4-oxidobenzoato-dilithium], [Li_2_(C_7_H_4_O_3_)(H_2_O)_2_]_*n*_, **1**, poly[tri­aqua-μ-4-oxidobenzoato-dilithium], [Li_2_(C_7_H_4_O_3_)(H_2_O)_3_]_*n*_, **2**, poly[μ-4-hy­droxy­benzoato-lithium], [Li(C_7_H_5_O_3_)]_*n*_, **3**, *catena*-poly[4-hy­droxy­benzoate [[di­aqua­sodium]-di-μ-aqua]], {[Na(H_2_O)_4_](C_7_H_5_O_3_)}_*n*_, **4**, poly[di-μ-aqua-aqua-μ-4-hy­droxy­benzoato-potassium], [K(C_7_H_5_O_3_)(H_2_O)_3_]_*n*_, **5**, poly[μ-aqua-μ-4-hy­droxy­benzoato-potassium], [K(C_7_H_5_O_3_)(H_2_O)]_*n*_, **6**, poly[aqua-μ-4-hy­droxy­benzoato-rubidium], [Rb(C_7_H_5_O_3_)(H_2_O)]_*n*_, **7**, poly[aqua-μ-4-hy­droxy­benzoato-caesium], [Cs(C_7_H_5_O_3_)(H_2_O)]_*n*_, **8**, poly[[μ-aqua-aqua­(μ-4-hy­droxy­benzoato)(4-hy­droxy­benzoic acid)sodium] monohydrate], {[Na(C_7_H_5_O_3_)(C_7_H_6_O_3_)(H_2_O)_2_]·H_2_O}_*n*_, **9**, poly[[(μ-4-hy­droxy­benzoato)(μ-4-hy­droxy­benzoic acid)rubidium] monohydrate], {[K(C_7_H_5_O_3_)(C_7_H_6_O_3_)]·H_2_O}_*n*_, **10**, and poly[[(μ-4-hy­droxy­benzoato)(μ-4-hy­droxy­benzoic acid)rubidium] monohydrate], {[Rb(C_7_H_5_O_3_)(C_7_H_6_O_3_)]·H_2_O}_*n*_, **11**.

## Abstract 2

Although 4-hy­droxy­benzoic acid (H_2_hba) is a relatively simple organic mol­ecule, it displays remarkable coordinative flexibility in its reactions with alkali metal hydroxides, forming ionic networks containing the dianion (hba^2−^), the monoanion (Hhba^−^) or the neutral acid species (H_2_hba). A common feature of the structures of the lattices is their layered arrangement: alternating hydro­philic layers made up of closely packed metal–oxygen polyhedra are separated by the hydro­phobic nonpolar com­ponents of the hy­droxy­benzoate linking units.

## Introduction   

In the study of chemistry, an understanding of the various types of chemical bonding is essential and thus it is not surprising that introductory chemistry courses at the secondary school level tend to have a strong emphasis on primary and secondary bonding. Many of the ideas that are presented to students have come from the analysis of bonds within and between mol­ecules, the structures of which have been determined by the technique of single-crystal X-ray diffraction. It is therefore somewhat surprising that the role of X-ray crystallography in providing detailed representations of mol­ecules is poorly recognized in many secondary school chemistry courses worldwide.

The reason that crystallography does not tend to form part of school chemistry curricula is perhaps due to the traditional inaccessibility of the technique. It would be fair to say that for a large part of the 20th century, crystal structures were determined by expert crystallographers who had extensive training in the technique and possessed a detailed understanding of the theory that underpinned the collection of data, the structural solution and the refinement process. For many chemists it was an unavailable technique unless one was able to collaborate with a crystallographer.

The 21st century has seen rapid development in both crystallographic hardware (sources and detectors) and software. Data sets can now be collected in minutes and improve­ments in both com­puters and crystallographic pro­grams with easy-to-use GUI inter­faces have allowed rapid structure solution and refinement of structures, particularly for routine structures. These advancements have allowed crys­tallographic novices to measure their own data and determine their structure quickly and with relative ease. Of course, there are traps for the inexperienced crystallographer, but nevertheless the technique of X-ray crystallography has never been so widely accessible.

As research chemists with a particular inter­est in crystallography and chemical education, we recognized an opportunity to expose secondary school students to crystallography. As part of a pilot elective program, students in the penultimate year of secondary education (Year 11; average age 16 years) from Scotch College, a school in the suburbs of Melbourne, were invited to participate in a seven-week after-school research investigation. The program consisted of weekly one hour sessions that included the basic principles of crystallography, workshops on the use of the *OLEX2* software package (Dolomanov *et al.*, 2009 ▸) and experimental work to make new crystalline com­pounds in the school laboratory. 
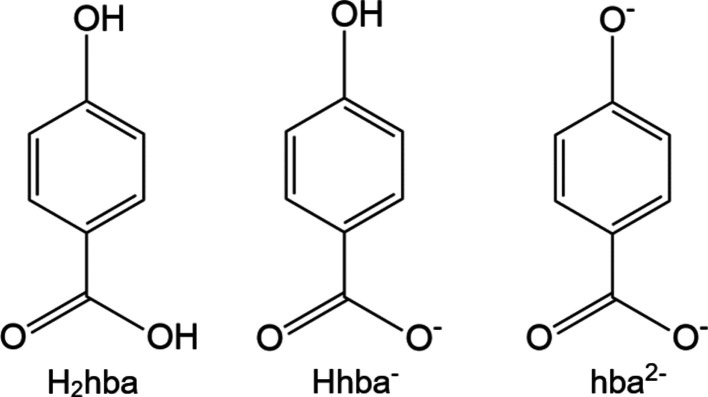


In order for school students to participate in a research project that involves synthesis and structure determination, we recognized that the synthetic work would need to be straightforward. Our research group has had a long-standing inter­est in coordination networks in which Zn^II^ and Co^II^ centres are linked by the dianion of 4-hy­droxy­benzoic acid (H_2_hba) (White *et al.*, 2015 ▸) and we thought an investigation of alkali metal salts of the acid might be easily performed.

The car­box­yl group of H_2_hba is deprotonated with relative ease (p*K*_a_ 4.5 com­pared with 4.2 for benzoic acid) to form the Hhba^−^ anion. Under some conditions, the phenolic group can also be deprotonated (p*K*_a_ 9.7 com­pared with 10.0 for phenol), to form the dianion hba^2−^. Given the coordinative versatility of the O-donor atoms of H_2_hba and its ability to form ions with either 1− and 2− charges, it was recognized that there may be an inter­esting systematic variation in the structures that could be obtained.

Transition-metal com­pounds of aromatic polycar­box­yl­ate ligands, such as benzene-1,4-di­car­box­yl­ate and benzene-1,3,5-tri­car­box­yl­ate, have been widely studied as a consequence of the potential applications for coordination polymers in drug delivery, gas storage, catalysis, separation and electrochemical applications (Li *et al.*, 2020 ▸; Shi *et al.*, 2019 ▸). On the other hand, relatively few car­box­yl­ate com­pounds of the *s*-block metals have been studied (Alnaqbi *et al.*, 2021 ▸; Banerjee & Parise, 2011 ▸) and phenolate/car­box­yl­ate ligands have received little attention.

The Cambridge Structural Database (CSD; Version 5.42, February 2021 release; Groom *et al.*, 2016 ▸) lists just four structures made only from H_2_hba and alkali metals. Skinner & Speakman (1951 ▸) isolated a potassium salt containing a proton placed symmetrically between adjacent car­box­yl­ate groups with a formula that can be represented as K(H_2_hba)(Hhba)·H_2_O. The structure of this com­pound is discussed further in this article and improved structural data with more accurate mol­ecular geometries are also provided. Skinner and Speakman mention the existence of an isostructural rubidium com­pound but no further details are given.

A sodium salt containing the anion Hhba^−^ has been made by reacting H_2_hba and sodium metal in tetra­hydro­furan (Dinnebier *et al.*, 1999 ▸). The salt can be represented by the formula Na(Hhba) and, using powder X-ray diffraction methods, the com­pound was shown to consist of layers of distorted NaO_6_ prisms with arene rings perpendicular to these layers and pointing up and down. The network is held together by hydrogen bonding between the phenolic hy­droxy groups.

Finally, a lithium metal oxide framework containing the hba^2−^ dianion of formula Li_2_(hba)(CH_3_OH)_2_ has been pre­pared by heating *t*-BuOLi and H_2_hba in a mixture of methanol and hexane (Zhao *et al.*, 2018 ▸). It is com­posed of parallel helical chains of Li—O rings. These chains are bridged by hba^2−^ units to form channels with a triangular cross section.

This article reports the structures of nine new alkali metal salts of H_2_hba and also provides data for the salt Rb(H_2_hba)(Hhba)·H_2_O, which has been mentioned in the literature but not characterized previously by single-crystal X-ray diffraction. The investigations that resulted in the structures described in this article were successful in generating inter­est and enthusiasm among the students who performed the experimental work and initial crystallographic processing, as well as enhancing their understanding of chemical bonding. Furthermore, we believe the research will be of inter­est to the wider scientific community. Not only are the structures of the individual com­pounds inherently inter­esting, but collectively they demonstrate the effects of reaction stoichiometry, ion size, hydrogen bonding and the nature of the ligand and solvent in the formation of ionic networks involving metal ions and organic anions.

## Experimental   

### Synthesis and crystallization   

In a series of reactions, H_2_hba was combined with the hydroxides of lithium, sodium, potassium, rubidium and caesium in different stoichiometric ratios.

Typically, this involved the addition of 0.10 g (0.73 mmol) of H_2_hba to the appropriate amount of metal hydroxide in 5 ml of warm water (50 °C). Crystals of the alkali metal salts suitable for single-crystal X-ray diffraction formed upon cooling and evaporation of the solvent.

Li_2_(hba)·2H_2_O and Li_2_(hba)·3H_2_O were formed from 2:1 stoichiometric ratios of LiOH and H_2_hba, whereas Li(Hhba) was prepared from a 1:1 reaction mixture. For sodium, a 1:1 mixture of the hydroxide and H_2_hba yielded Na(Hhba)(H_2_hba)·3H_2_O and a 1:1.5 mixture yielded Na(Hhba)·4H_2_O.

A 1:1 combination of KOH and H_2_hba yielded both plate-shaped crystals of K(Hhba)·3H_2_O and rod-shaped crystals of K(Hhba)·H_2_O, whereas K(H_2_hba)(Hhba)·H_2_O crystals were obtained from a 1:2 reaction mixture. Crystals of Rb(H_2_hba)(Hhba)·H_2_O were formed from a 1:1 mixture, and a 1:2 mixture yielded Rb(Hhba)·H_2_O. Cs(Hhba)·H_2_O was formed in reactions using different stoichiometric ratios (the crystal used for data collection came from a 1:2.5 CsOH–H_2_hba mixture).

### Refinement   

Crystal data, data collection and structure refinement details are summarized in Table 1 ▸. The H atoms of the water mol­ecules, phenolic groups and carb­oxy­lic acid groups were located in difference Fourier maps and refined with O—H distances restrained to 0.85 Å, except for the H atoms involved in the short strong hydrogen bonds in K(H_2_hba)(Hhba)·H_2_O and Rb(H_2_hba)(Hhba)·H_2_O, which were located in difference Fourier maps and refined independently. The *U*_iso_ values of the H atoms bonded to O atoms were allowed to refine. Other H atoms were placed in calculated positions and refined as riding atoms, with C—H = 0.95 Å and *U*_iso_(H) = 1.2*U*_eq_(C) for aromatic H atoms. The uncoordinated water mol­ecules are disordered in com­pound **10** and the H atoms of the water mol­ecules are disordered in both **10** and **11**. As a consequence, their positions have not been assigned. The phenolic H atoms in **10** and **11** are disordered over two positions. Details of the refinements can be found in the embedded instruction files in the CIF files.

## Results and discussion   

Each of the com­pounds formed by the reaction of the alkali metal hydroxides and H_2_hba in aqueous solution can be classified within one of three categories according to the metal–H_*n*_hba ratio in the crystal structure (*n* = 0, 1 or 2).

**Type I:** *M*_2_(hba)(H_2_O)_*x*_ (*M* = Li, *x* = 2 and 3).

**Type II:** *M*(Hhba)(H_2_O)_*x*_ (*M* = Li, *x* = 0; *M* = Na, *x* = 4; *M* = K, *x* = 3; *M* = K, Rb or Cs, *x* = 1).

**Type III:** Na(Hhba)(H_2_hba)(H_2_O)_2_·H_2_O and *M*(Hhba)(H_2_hba)·H_2_O (*M* = K or Rb).

### Structure description of type I com­pounds: *M*_2_(hba)(H_2_O)_*x*_   

Whilst H_2_hba can lose protons to form either the 1− or 2− ions, *i.e.* Hhba^−^ or hba^2−^, lithium is the only group I metal that yielded salts containing the dianion under the reaction conditions employed in this investigation. The loss of the weakly acidic phenolic proton in the presence of the lithium ion may be a consequence of the relatively small size of the Li^+^ ion resulting in a strong Li—O inter­action, which in turn promotes the loss of the proton of the hy­droxy group.

**Figure 1:**
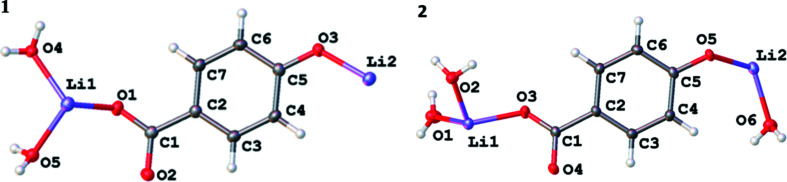
The asymmetric units of Li_2_(hba)(H_2_O)_2_ (**1**) and Li_2_(hba)(H_2_O)_3_ (**2**), showing the atom-labelling schemes. Displacement ellipsoids are drawn at the 50% probability level. H atoms are represented by spheres of arbitrary size.

A dihydrate, **1**, and a trihydrate, **2**, crystallized from aqueous 2:1 molar mixtures of LiOH and H_2_hba. The structures of their asymmetric units are shown in Fig. 1 ▸.

**Figure 2:**
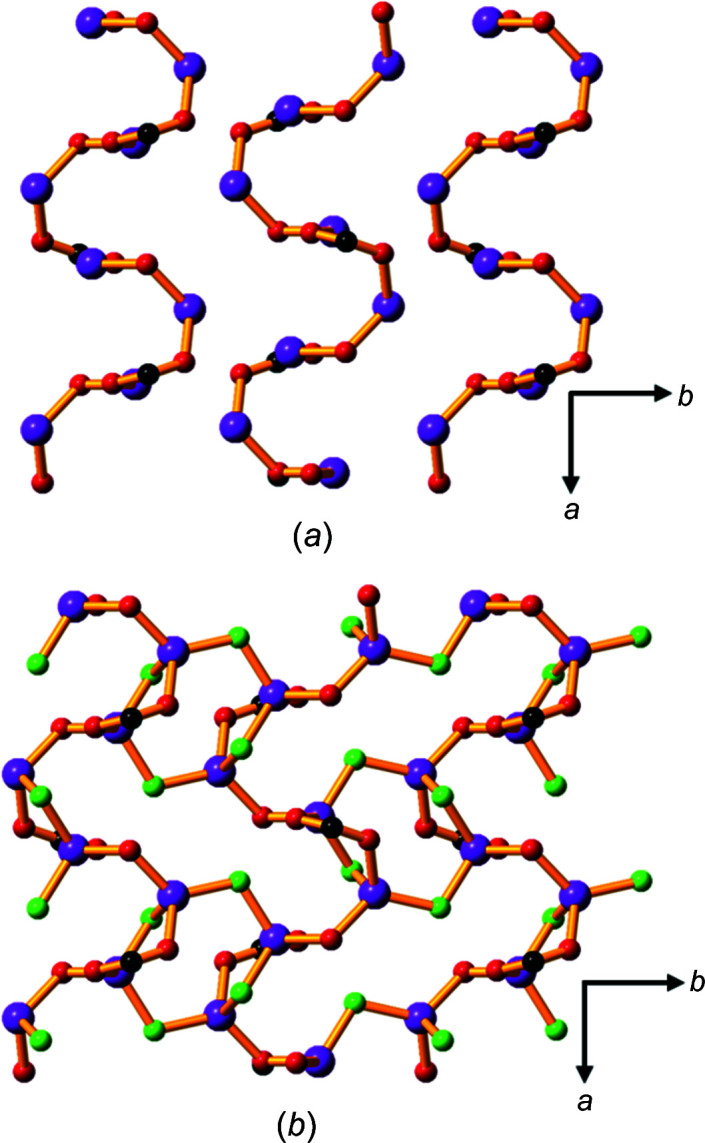
The Li—O layers in com­pound **1** showing (*a*) lithium centres linked by car­box­yl­ate and phenolate O atoms to form helical chains within the Li—O layers, and (*b*) the same layer as in (*a*), but with bridging water mol­ecules included. The H and C atoms of the arene rings have been omitted. Colour code: Li purple, car­box­yl­ate and phenolate O red, water O green and C black.

Compound **1** contains Li—O layers (hydro­philic sheets) that are formed from lithium ions linked by car­box­yl­ate and phenolate groups from hba^2−^, creating helical chains which run in the *a* direction (Fig. 2 ▸ *a*). Bridging water mol­ecules (shown in green in Fig. 2 ▸ *b*) link these chains to form two-dimensional (2D) layers. Each Li^+^ ion is four-coordinate and bonded to two bridging water mol­ecules, a bridging phenolate O atom and a car­box­yl­ate O atom.

**Figure 3:**
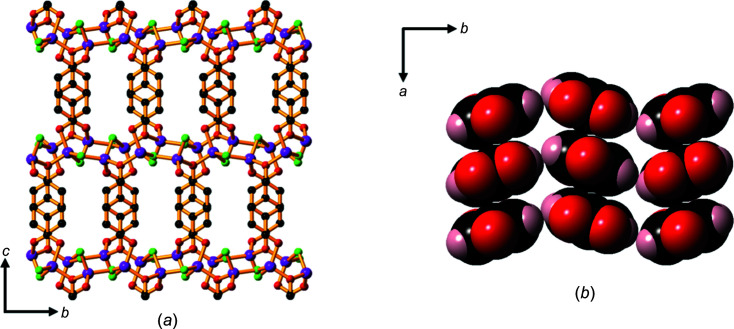
The structure of **1**, showing (*a*) a view down the *a* axis, highlighting the hydro­phobic pillars of hba^2−^ units between the hydro­philic Li—O sheets (H atoms have been omitted), and (*b*) a space-filling model of the closely packed hba^2−^ anions arranged in parallel and anti­parallel configurations. Colour code: Li purple, C black, car­box­yl­ate and phenolate O red, water O green and H pale pink.

The extended packing arrangement in **1** is shown in Fig. 3 ▸(*a*). The hba^2−^ ligands extend above and below the Li—O sheets to form a pillared-type three-dimensional (3D) network. The hydro­philic sheets are separated by the hydro­phobic sections of hba^2−^, with a sheet-to-sheet separation of approximately 9.3 Å (half the length of the *c* axis). Hydro­philic *M*—O layers separated by hydro­phobic regions is an arrangement common to all of the structures of the alkali metal–H_*n*_hba com­pounds described in the current work. This layered architecture is characteristic of many of the structures pre­viously reported for coordination polymers of alkali metals (Banerjee & Parise, 2011 ▸).

The hba^2−^ pillars form stacks arranged in a face-to-face pattern (Fig. 3 ▸ *b*), with alternating orientations of the ligand.

**Figure 4:**
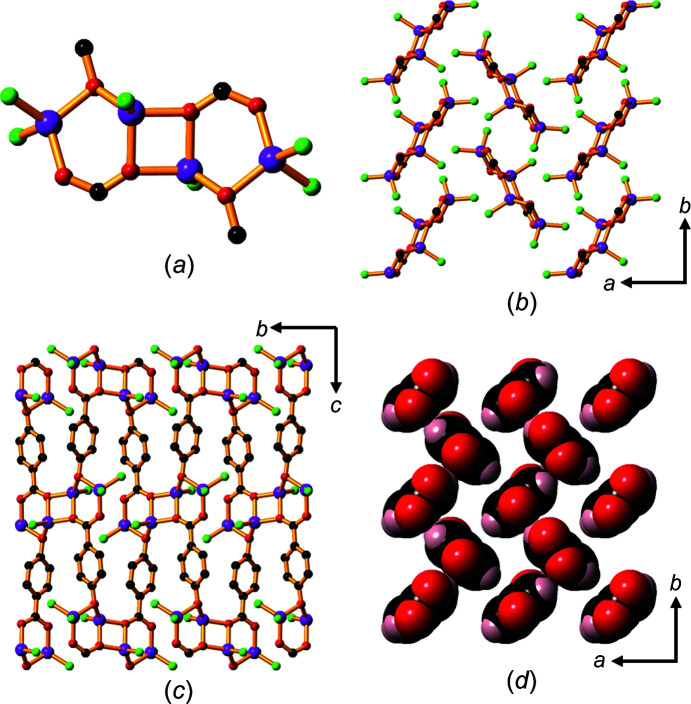
The structure of **2**, showing (*a*) Li_4_ units containing four- and six-membered rings, (*b*) Li_4_ units within a hydro­philic sheet, (*c*) neighbouring Li_4_ units linked to each other in adjacent sheets that are separated by the hydro­phobic sections of hba^2−^ linkers and (*d*) the hba^2−^ units packed in an edge-to-face pattern. H atoms have been omitted in parts (*a*), (*b*) and (*c*). Colour code: Li purple, car­box­yl­ate and phenolate O red, water O green, C black and H pale pink.

Whilst the trihydrate, **2**, is also com­posed of hydro­philic Li—O sheets separated by hydro­phobic organic regions, there are marked differences in its structure com­pared with the structure of **1**. Each car­box­yl­ate O atom is bonded to one Li^+^ ion in com­pound **1**, whereas one of the car­box­yl­ate O atoms is bonded to two metal ions in **2**, forming a 2D network in which four-coordinate lithium centres form discrete intra­sheet Li_4_ units within a sheet involving four- and six-membered rings (Fig. 4 ▸ *a*).

Fig. 4 ▸(*b*) shows the arrangement of these Li_4_ units within a hydro­philic sheet. Although the Li_4_ units within each sheet are not linked by strong bonds, neighbouring Li_4_ units are linked to other Li_4_ units *via* bonds to atoms in the adjacent hydro­philic sheets, resulting in a 2D network which extends in the *bc* plane (Fig. 4 ▸ *c*). The sheet-to-sheet separation is about 9.15 Å (half the length of the *c* axis). Unlike the dihydrate salt, **1**, the hba^2−^ pillars are packed in an edge-to-face arrangement, inverted along the *a* axis, as shown in Fig. 4 ▸(*d*).

### Structure description of type II com­pounds: *M*(Hhba)(H_2_O)_*x*_   

**Figure 5:**
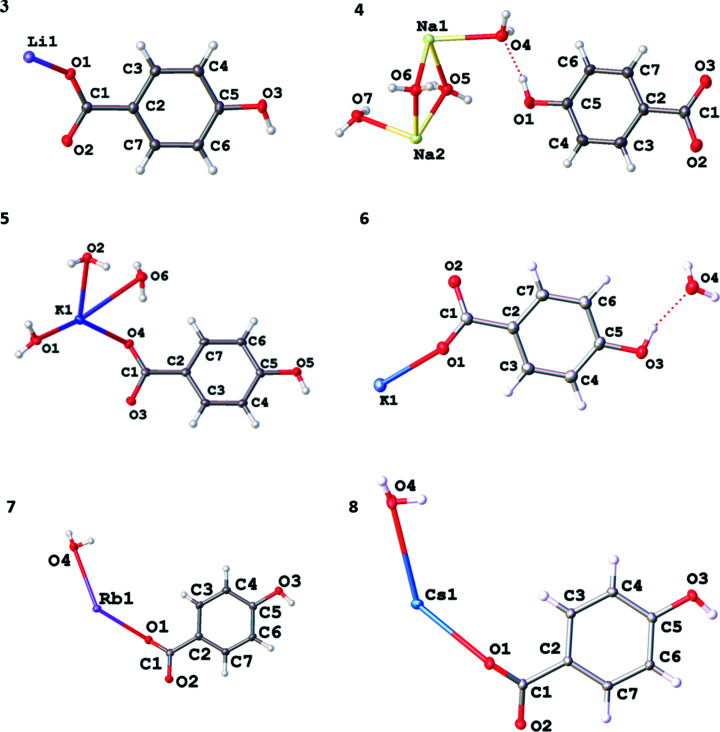
The asymmetric units of Li(Hhba), **3**, [Na(H_2_O)_4_][Hhba], **4**, K(Hhba)(H_2_O)_3_, **5**, K(Hhba)(H_2_O), **6**, Rb(Hhba)(H_2_O), **7**, and Cs(Hhba)(H_2_O), **8**, showing the atom-labelling schemes. Displacement ellipsoids are drawn at the 50% probability level. H atoms are represented by spheres of arbitrary size. The red dotted lines represent hydrogen-bonding inter­actions.

All the alkali metals in this study form com­pounds con­taining the monoanion Hhba^−^ and have the general formula *M*(Hhba)(H_2_O)_*x*_ (*M* = Li, *x* = 0; *M* = Na, *x* = 4; *M* = K, *x* = 3; *M* = K, Rb or Cs, *x* = 1). Their asymmetric units are shown in Fig. 5 ▸. Four different structural arrangements are observed in this group of salts.

**Figure 6:**
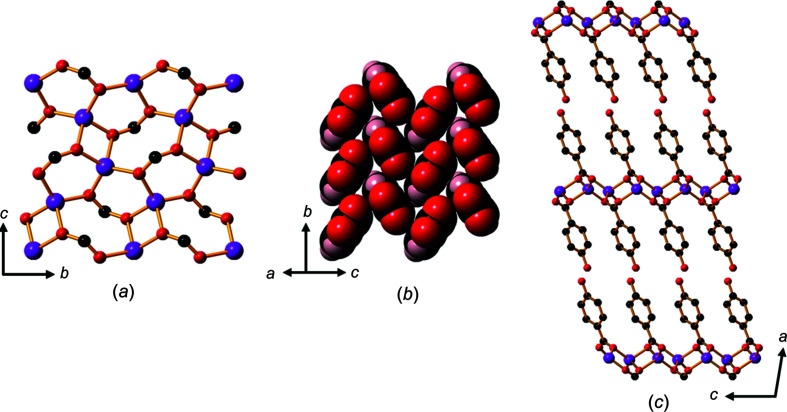
The structure of **3**, showing (*a*) four-, six- and eight-membered rings formed by tetra­hedral lithium centres and car­box­yl­ate atoms, (*b*) the Hhba^−^ ligands closely packed in a face-to-edge arrangement and (*c*) a packing diagram of com­pound **3** viewed along the *b* axis. H atoms have been omitted in parts (*a*) and (*c*). Colour code: Li purple, O red, C black and H pale pink.

Compound **3**, LiHhba, is a 2D ionic network formed in a 1:1 reaction of LiOH and H_2_hba. Within the Li—O layers, each car­box­yl­ate O atom bridges two lithium ions to form four-, six- and eight-membered rings (Fig. 6 ▸ *a*). The Hhba^−^ pillars of each layer are closely stacked in an edge-to-face pattern (Fig. 6 ▸ *b*), with each Li—O layer about 14.9 Å apart (the length of the *a* axis).

Each phenolic OH group forms two hydrogen bonds with phenolic OH groups on an adjacent 2D framework (Fig. 6 ▸ *c*), holding the hydro­phobic regions from the two layers together in a bilayer motif. There are, therefore, two types of hydro­philic layers within the structure: the layers containing Li^+^ ions and, between these layers, ones com­posed of phenolic OH groups. The presence of a bilayer packing motif has been observed in many other metal car­box­yl­ates, although it is more common in aliphatic salts than in salts of aromatic acids (Vela & Foxman, 2000 ▸).

Dinnebier *et al.* (1999 ▸) reported the synthesis of a salt of formula Na(Hhba) which has a similar bilayer structure to that of Li(Hhba) described above. It was made by reacting H_2_hba and sodium metal in tetra­hydro­furan and powder X-ray diffraction was used to determine its structure. The salt consists of layers of distorted NaO_6_ prisms and the layers are held together by hydrogen bonding between the phenolic groups. Unlike Li(Hhba), the arene rings, which are orientated perpendicular to these layers, are arranged in parallel stacks.

**Figure 7:**
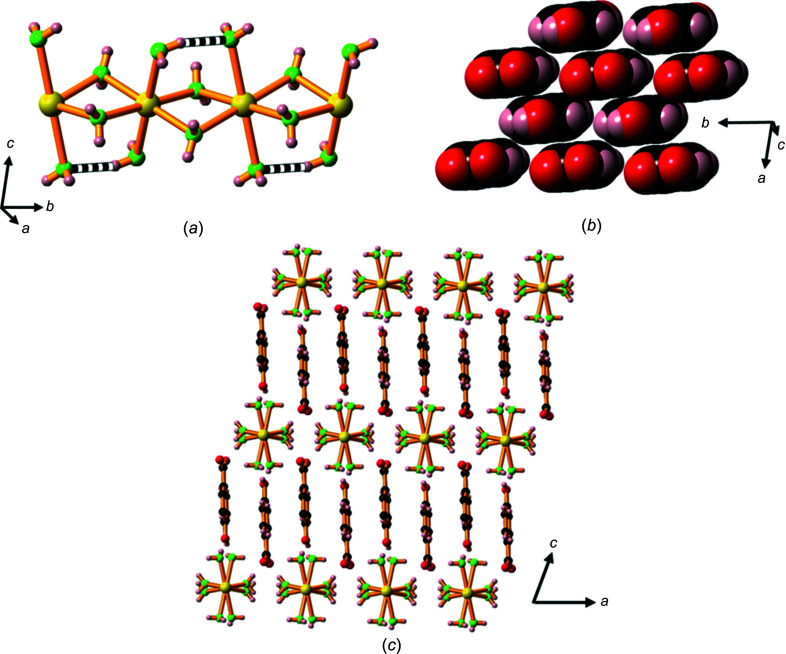
The structure of **4**, showing (*a*) {Na(H_2_O)_4_^+^}_*n*_ chains involving bridging water mol­ecules; intra­chain hydrogen bonding between pairs of axial water mol­ecules (shown as black and white bonds) ‘pinches’ the O atoms in these mol­ecules together in pairs. (*b*) The anti­parallel slipped stacking pattern of Hhba^−^ anions and (*c*) the packing arrangement, viewed along the *b* axis. Colour code: Na yellow, car­box­yl­ate and phenolate O red, water O green, C black and H pale pink.

The structure of **4**, [Na(H_2_O)_4_][Hhba], is quite different to all the other structures described in this article because the metal centres are not bonded to organic anions. The Na^+^ ions are present as {Na(H_2_O)_4_^+^}_*n*_ chains in which octa­hedral Na^+^ ions are located within a square-planar arrangement of four bridging water mol­ecules (Fig. 7 ▸ *a*). Strong intra­chain hydrogen bonding [O⋯O = 2.696 (2) Å] involving the axial water mol­ecules ‘pinches’ the O atoms of the water mol­ecules together in pairs.

The Hhba^−^ pillars are arranged in an anti­parallel slipped stacking pattern (Fig. 7 ▸ *b*). They form hydrogen bonds with the water mol­ecules coordinated to the Na^+^ ions, with a layer-to-layer separation of approximately 12.2 Å (Fig. 7 ▸ *c*).

**Figure 8:**
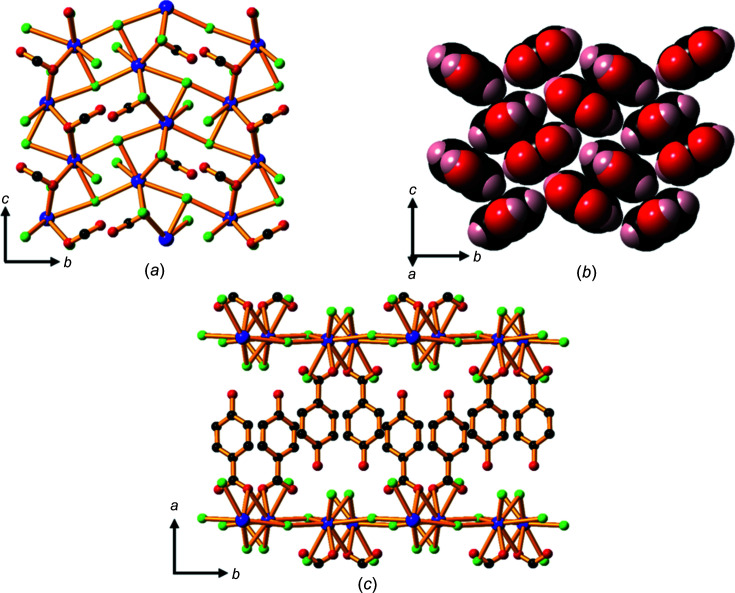
The structure of **5**, showing (*a*) layers of distorted KO_8_ prisms formed from the metal ions, the O atoms of water mol­ecules (shown in green) and one car­box­yl­ate O atom from each Hhba^−^ unit. (*b*) The edge-to-face and face-to-face stacking of the Hhba^−^ ligands; note the inversion that occurs within face-to-face pairs. (*c*) The packing arrangement, viewed along the *c* axis. H atoms have been omitted in parts (*a*) and (*c*). Colour code: K blue, car­box­yl­ate and phenolate O red, water O green, C black and H pale pink.

The potassium salt **5**, K(Hhba)(H_2_O)_3_, may be considered to be a 2D network. The hydro­philic K—O layer shown in Fig. 8 ▸(*a*) is com­posed of distorted KO_8_ square anti­prisms formed between the metal ions and the O atoms from water mol­ecules and one car­box­yl­ate O atom of each Hhba^−^ unit bridging K^+^ centres. Fig. 8 ▸(*b*) shows the face-to-face and edge-to-edge close packing of the organic pillars; π–π stacking inter­actions are present between the cofacial Hhba^−^ ligands [the centroids of the face-to-face pairs are 3.503 (2) Å apart]. The arene rings of Hhba^−^ are perpendicular to the hydro­philic K—O layers and point up and down; they are inter­leaved with the arene rings of adjacent layers, as shown in Fig. 8 ▸(*c*). Phenolic OH groups participate in hydrogen-bonding inter­actions with the water mol­ecules bonded to the K centres in adjacent parallel sheets, which are about 12.3 Å apart.

**Figure 9:**
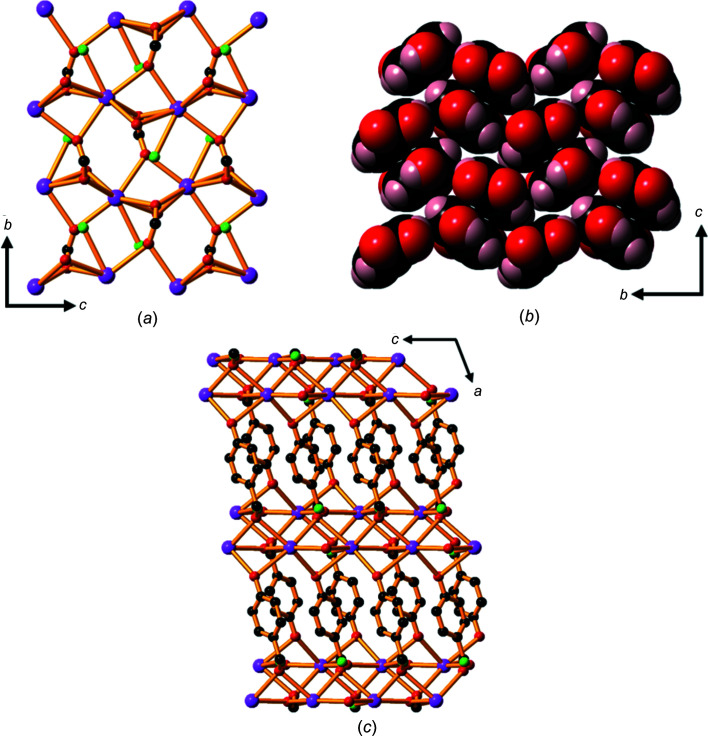
The structure of **7**, showing (*a*) the Rb—O layer, viewed along the *a* axis; C atoms in the arene rings and H atoms have been omitted. (*b*) The stacking of the Hhba^−^ ligands, similar to that seen in com­pound **5**; the centroids of the face-to-face pairs are approximately 3.83 Å apart. (*c*) The packing arrangement, viewed down the *b* axis, with H atoms omitted. Colour code: Rb purple, car­box­yl­ate and phenolate O red, water O green, C black and H pale pink.

3D networks with the general formula *M*(Hhba)(H_2_O) are formed with potassium, rubidium and caesium. Compound **6** is ortho­rhom­bic and shares some structural features with the isostructural monoclinic com­pounds, **7** and **8**. Taking the rubidium salt, com­pound **7**, as the exemplar, each Rb^+^ ion is seven-coordinate and each car­box­yl­ate group is linked to four Rb^+^ ions (Fig. 9 ▸ *a*). The phenolic O atom, even though pro­tonated, bridges two Rb^+^ ions. Each water mol­ecule bonds to only one metal ion. The Hhba^−^ pillars are packed in a face-to-face and edge-to-edge arrangement (Fig. 9 ▸ *b*), forming the 3D network shown in Fig. 9 ▸(*c*).

As seen for the rubidium salt in Fig. 9 ▸(*b*), there is a pronounced rotation of the atoms in the car­box­yl­ate groups away from the plane of the aromatic ring in the potassium, rubi­dium and caesium *M*(Hhba)(H_2_O) com­pounds [K 25.13 (8), Rb 26.86 (8) and Cs 24.49 (6)°]. The metal cations bonded to the car­box­yl­ate O atoms in these com­pounds are also not in the plane of the car­box­yl­ate group. Such configurations are uncommon in transition-metal–car­box­yl­ate com­plexes because the transition-metal ion is generally located in the plane of the car­box­yl­ate group. In *s*-block com­pounds, the bonds are mainly ionic in nature with little or no directionality and thus crystal packing forces and other steric considerations can dominate.

The structures of **6**, **7** and **8** have similar connectivity and are com­pared in Fig. S1 of the supporting information.

### Structure description of type III com­pounds: Na(Hhba)(H_2_hba)(H_2_O)_2_·H_2_O and *M*(Hhba)(H_2_hba)·H_2_O   

**Figure 10:**
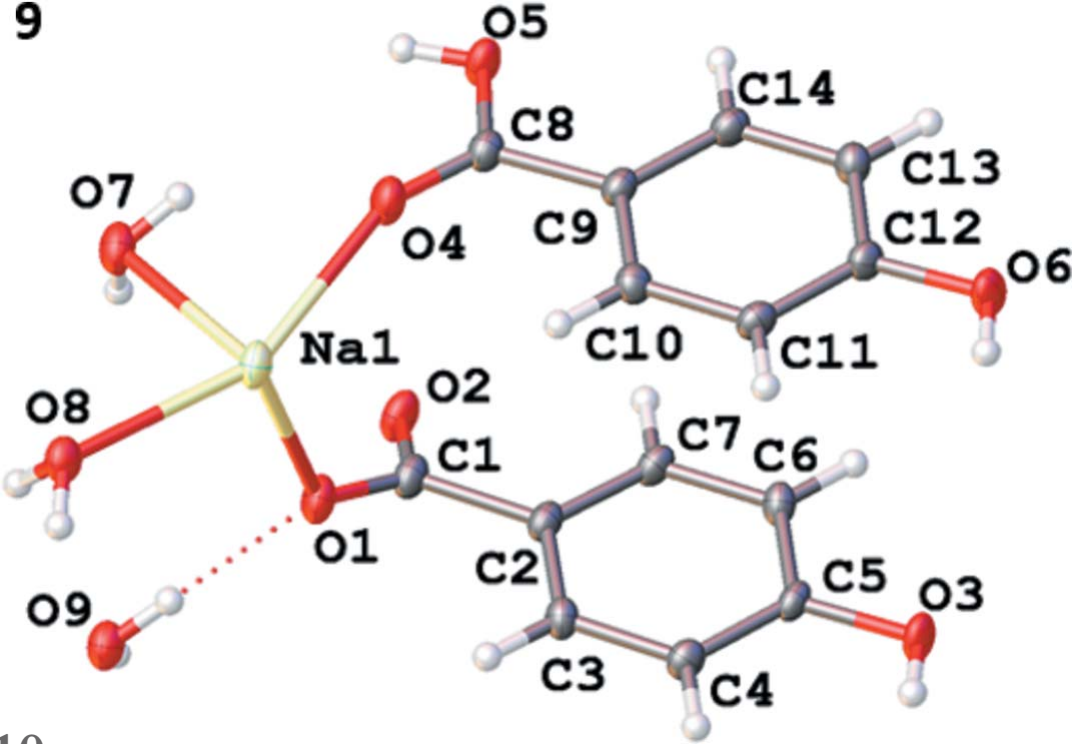
The asymmetric unit of Na(Hhba)(H_2_hba)(H_2_O)_2_·H_2_O, **9**, showing the atom-labelling scheme. Displacement ellipsoids are drawn at the 50% probability level. H atoms are represented by spheres of arbitrary size. The red dotted line represents a hydrogen-bonding inter­action.

Na(Hhba)(H_2_hba)(H_2_O)_2_·H_2_O (com­pound **9**) is a 2D layer lattice which, like the previous com­pounds, is com­posed of hydro­philic and hydro­phobic regions. The hydro­phobic regions contain both neutral H_2_hba and the monoanion, Hhba^−^, both of which are coordinated to each metal ion (Fig. 10 ▸).

**Figure 11:**
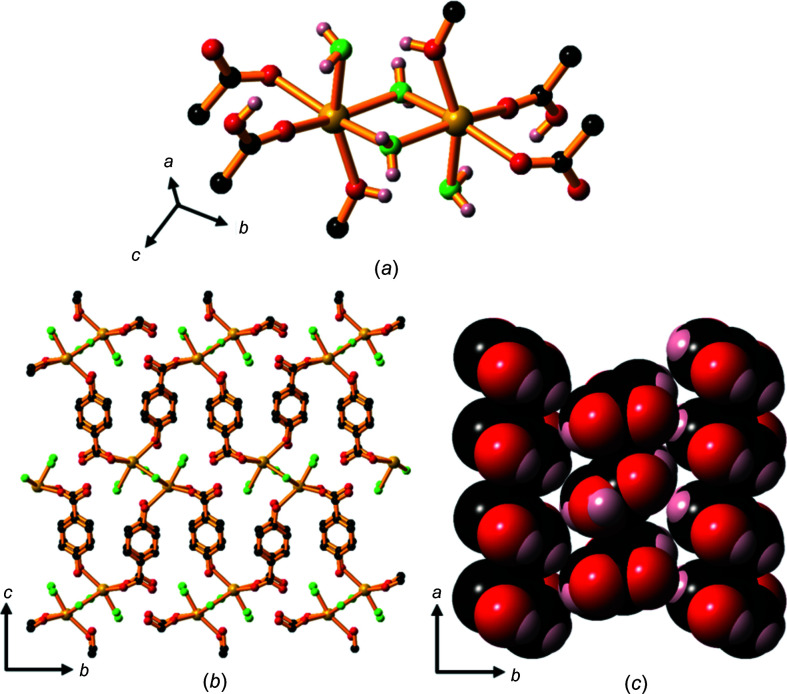
The structure of **9**, showing (*a*) a disodium unit linked by a pair of bridging water mol­ecules, (*b*) the packing arrangement, viewed down the *a* axis (H atoms have been omitted), and (*c*) the close-packed alternating stacking of the H_2_hba and Hhba^−^ ligands. Colour code: Na yellow, car­box­yl­ate and phenolate O red, water O green, C black and H pale pink.

There is extensive hydrogen bonding in each Na—O layer and the Na^+^ ions are linked by a pair of bridging water mol­ecules to form disodium units (Fig. 11 ▸ *a*). Octa­hedral Na^+^ ions are coordinated by three water mol­ecules, two bridging and one terminal. The Na^+^ ion is also coordinated by one phenolic O atom; the phenolic group on the other organic ligand is noncoordinated and does not participate in hydrogen bonding. Finally, the remaining *cis* sites on each Na^+^ ion are occupied by an O atom of a protonated car­box­ylic acid group and an O atom of a deprotonated car­box­yl­ate group.

The disodium units are linked by the organic ligands to form a 2D network in the *bc* plane. Within each network, the organic units separate the Na^+^ ions by approximately 9.7 Å, as shown in Fig. 11 ▸(*b*). Hydrogen bonds to intra­planar uncoordinated water mol­ecules and between coordinated water mol­ecules and phenolic groups on adjacent layers link the layers together. Alternate stacks of H_2_hba and Hhba^−^ ligands are shown in Fig. 11 ▸(*c*).

Potassium and rubidium hydroxide react with H_2_hba to form com­pounds **10** and **11** with a general formula that can be represented as *M*(Hhba)(H_2_hba)·H_2_O. As indicated in the *Introduction*, the structure of the potassium salt was first determined by Skinner & Speakman (1951 ▸) and a more accurate study was performed in 1968 (Manojlović, 1968 ▸). As part of our investigation, single-crystal X-ray diffraction of a crystal of this com­pound has confirmed the previous results and provides more accurate mol­ecular geometries. Furthermore, we have also obtained the first single-crystal X-ray diffraction data for the isostructural rubidium analogue that is mentioned in the article of Skinner and Speakman.

**Figure 12:**
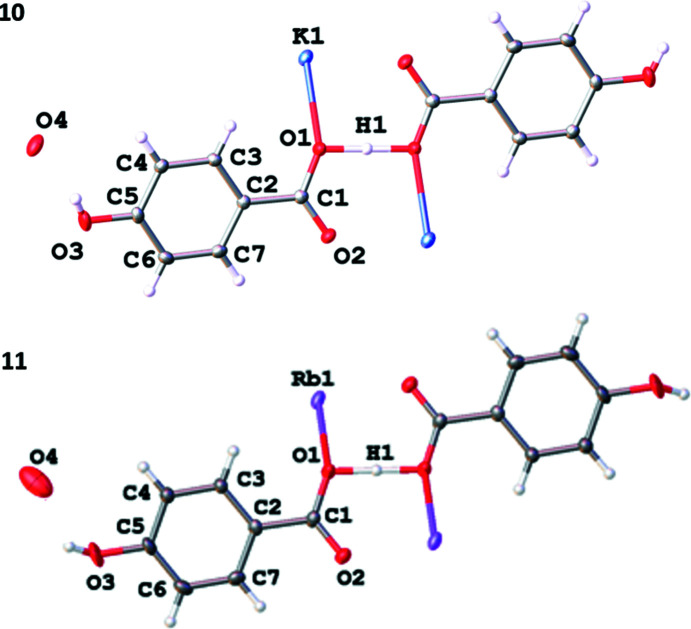
The asymmetric units of K(Hhba)(H_2_hba)·H_2_O, **10**, and Rb(Hhba)(H_2_hba)·H_2_O, **11**, expanded to show the short strong hydrogen bonds present between car­box­yl­ate O atoms. Displacement ellipsoids of atoms other than hydrogen are drawn at the 50% probability level. H atoms are represented by spheres of arbitrary size. In the case of both **10** and **11**, only one position of a disordered phenolic H atom is shown for clarity. The water mol­ecule in **11** is disordered over two positions; the H atoms on O4 are disordered in both com­pounds and were not assigned.

A feature of these potassium and rubidium com­pounds is the presence of an unusually strong inter­action between two car­box­yl­ate O atoms involving a three-centre four-electron O⋯H⋯O hydrogen bond (Fig. 12 ▸). The O atoms involved are closely separated: 2.448 (3) Å in the potassium com­pound and 2.466 (4) Å in the rubidium com­pound. These bonds can be described as ‘short strong’ (SSHB) or ‘low barrier’ (LBHB) hydrogen bonds and may be considered partly covalent in character (Reiersølmoen *et al.*, 2020 ▸; Saunders *et al.*, 2019 ▸). On the basis of a single peak of electron density, we have elected to assign the H atom to the mid-point of the two O atoms. However, it is possible that the H atom is disordered over two closely separated positions. The crystallographic data does not allow us to clearly differentiate between a symmetrical or a disordered model.

Although we have chosen to represent the formulae of these com­pounds as *M*(Hhba)(H_2_hba)·H_2_O, if the proton is fixed in the symmetrical position, the organic ligand in these com­pounds could be regarded as the fusion of a H_2_hba and a Hhba^−^ unit and represented as H(Hhba)_2_^−^, with each hy­droxy­benzoate unit having an average charge of −0.5.

**Figure 13:**
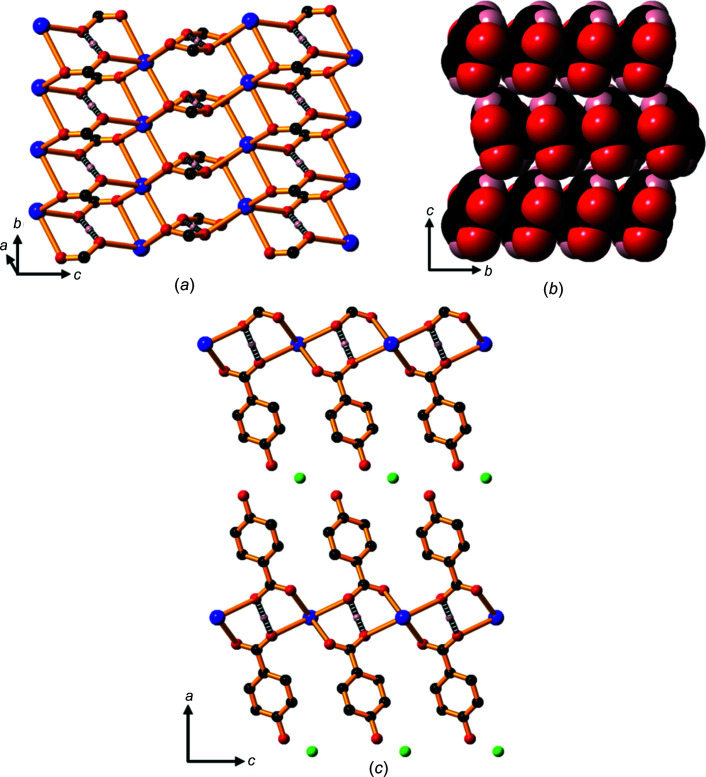
(*a*) The structure of **10**, showing the K—O layer, indicating the bridging car­box­yl­ate units and the location of the O⋯H⋯O SSHB inter­actions (shown as black and white bonds); H and C atoms in the arene rings have been omitted. (*b*) The organic ligands arranged in parallel stacks and (*c*) the packing arrangement, viewed down the *b* axis, showing the bilayer motif; H atoms on the arene rings and water mol­ecules have been omitted. For clarity, the disorder in the water mol­ecules located between adjacent hydro­phobic regions is not shown. Colour code: K blue, car­box­yl­ate and phenolate O red, water O green, C black and H pale pink.

The com­pounds form 2D ionic networks with both car­box­yl­ate O atoms bridging six-coordinate metal centres (Fig. 13 ▸ *a*). The hy­droxy­benzoate pillars are closely packed in parallel stacks in a face-to-face and edge-to-edge pattern, as shown in Fig. 13 ▸(*b*).

Hydrogen bonding between the phenolic OH groups and un­coordinated water mol­ecules located between two adjacent hydro­phobic regions holds the lattice together and creates a bi­layer motif with a hydro­philic layer of water mol­ecules and phe­nolic OH groups midway between the *M*—O hydro­philic layers (Fig. 13 ▸ *c*). The *M*—O layers are about 16.4 Å apart.

The intra­layer water mol­ecules are disordered in com­pound **10** and the H atoms of the water mol­ecules are disordered in both com­pounds **10** and **11**, and their positions have not been assigned. The phenolic H atoms are disordered over two positions.

### Packing of hydro­phobic and hydro­philic layers   

As described above, a common feature of the structures of the alkali metal salts of H_2_hba described in this work is the hydro­philic *M*—O layers separated by hydro­phobic regions of hy­droxy­benzoate units. It is perhaps surprising that similar layer structures are obtained regardless of the level of protonation of the hy­droxy­benzoate unit, *i.e.* the fully pro­ton­ated H_2_hba, the monoanion Hhba^−^ and the dianion hba^2−^ are arranged in such a way that hydro­philic groups participate in hydrogen bonding which appears to be important in the generation of layered structures. The hy­droxy­benzoate units adopt different packing arrangements in this series of com­pounds, including face-to-face, edge-to-face and a mixture of both. A slipped stacking arrangement is observed in com­pound **4** and three com­pounds contain a bilayer motif in which two discrete hydro­phobic layers are held together by hydro­gen bonds (com­pounds **3**, **10** and **11**).

Although the packing modes differ, the number of hy­droxy­benzoate ligands that pass through a cross-sectional plane through the hydro­phobic layers and parallel to the *M*—O hydro­philic layer is calculated to be between 4.6 to 5.2 units per nm^2^ for most com­pounds. As indicated in the space-filling representations shown above, ligands are packed closely, with distances between adjacent mol­ecules close to or less than that expected based on the van der Waals radii of their constituent atoms. The organic units in com­pound **2** are less closely packed (3.8 ligands per nm^2^) due to penetration of coordinated water mol­ecules into the ‘hydro­phobic’ layers.

Close packing of the organic ligands seems to be a dominant factor in determining overall structure. As the ratio of metal ions, coordinated water mol­ecules and hy­droxy­benzoate units changes in the com­pounds, the almost constant packing density of the hy­droxy­benzoate units influences the geometry and connectivity of the *M*—O hydro­philic layer.

**Figure 14:**
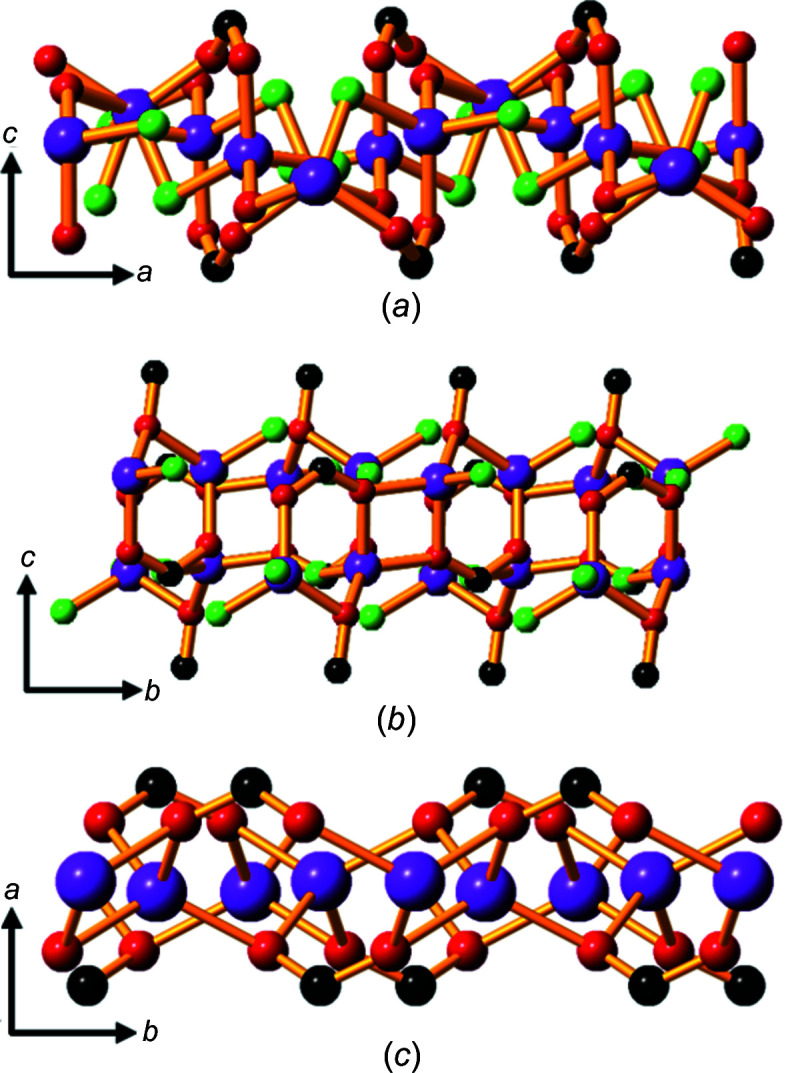
The hydro­philic layer in the lithium salts: (*a*) sinusoidal pattern of Li^+^ ions in com­pound **1**, (*b*) two planes of Li^+^ ions in **2** and (*c*) a single plane of Li^+^ ions in **3**. H atoms have been omitted. Colour code: Li purple, water O green, car­box­yl­ate and phenolate O red and C black.

In com­pounds **3**, **4**, **5**, **10** and **11**, the metal ions are in, or almost in, a plane and in com­pound **1** a sinusoidal pattern of metal ions is observed. Two planes of metal ions are present within each hydro­philic layer in com­pounds **2**, **6**, **7**, **8** and **9**, an arrangement that allows the metal ions to be packed more densely in the layer than if only a single plane of metal ions were present. Fig. 14 ▸ shows the different topologies of the hydro­philic layers in the three lithium salts.

## Conclusion   

Given that H_2_hba is a relatively simple organic mol­ecule, the salts that crystallize from aqueous solution when H_2_hba reacts with alkali metal hydroxides might be expected to form only a few different structures. However, although the structures of the salts have features in common, they are also remarkably different. The ligand can bond to metal ions as a dianion (hba^2−^), a monoanion (Hhba^−^) or as the neutral acid species (H_2_hba), allowing for considerable possible structural variation.

Most of the salts are either 2D or 3D ionic networks com­posed of alternating hydro­philic layers of closely packed *M*—O polyhedra separated by the hydro­phobic nonpolar com­ponent of the pillar-like hy­droxy­benzoate linking units.

The hy­droxy­benzoate units in the hydro­phobic sections of the lattices are usually closely packed; a feature that seems to impact on the arrangement of metal ions in the hydro­philic layers and hence the structures overall. Whilst the ligands are usually present in two distinct orientations within a hydro­phobic layer [with the exception of *M*(Hhba)(H_2_hba)·H_2_O; *M* = K or Rb], the packing observed in different salts includes edge-to-face, face-to-face and a mixture of the two. A bilayer packing arrangement is formed in three com­pounds. The metal ions may be both in the plane and out of the plane of the coordinating car­box­yl­ate group. The car­box­yl­ate group generally remains in the plane of the arene rings, although significant twisting is noted in *M*(Hhba)(H_2_O) (*M* = K, Rb or Cs).

Hydrogen bonds play a key role in the structure of all the com­pounds. They are present between the hydro­philic layers, as in K(Hhba)(H_2_O)_3_, within layers, as in Li_2_(hba)(H_2_O)_2_, and also in the form of an SSHB in *M*(Hhba)(H_2_hba)·H_2_O (*M* = K or Rb).

Li^+^ is the only metal ion to give the dianion form of the ligand under the reaction conditions used in this investigation, perhaps as a consequence of its high charge density and the strength of the Li—O inter­action.

This investigation of the alkali metal salts of H_2_hba proved to be highly successful in demonstrating the fundamental roles of strong and weak bonding inter­actions in the structure of materials to senior secondary school students. The salts display bonding types ranging from covalent bonds, ionic attractions, ion–dipole inter­actions, neutral and charge-assisted hydrogen bonding, and dispersion forces. Whilst the salts are readily synthesized, the structures of most of the com­pounds prepared in this study have not been reported previously, allowing students to experience genuine scientific discovery and also to appreciate the power, precision and convenience of the technique of X-ray crystallography for structure analysis.

## Supplementary Material

Crystal structure: contains datablock(s) compound1, compound2, compound3, compound4, compound5, compound6, compound7, compound8, compound9, compound10, compound11, global. DOI: 10.1107/S2053229621005465/ep3017sup1.cif

Structure factors: contains datablock(s) compound1. DOI: 10.1107/S2053229621005465/ep3017compound1sup2.hkl

Structure factors: contains datablock(s) compound2. DOI: 10.1107/S2053229621005465/ep3017compound2sup3.hkl

Structure factors: contains datablock(s) compound3. DOI: 10.1107/S2053229621005465/ep3017compound3sup4.hkl

Structure factors: contains datablock(s) compound4. DOI: 10.1107/S2053229621005465/ep3017compound4sup5.hkl

Structure factors: contains datablock(s) compound5. DOI: 10.1107/S2053229621005465/ep3017compound5sup6.hkl

Structure factors: contains datablock(s) compound6. DOI: 10.1107/S2053229621005465/ep3017compound6sup7.hkl

Structure factors: contains datablock(s) compound7. DOI: 10.1107/S2053229621005465/ep3017compound7sup8.hkl

Structure factors: contains datablock(s) compound8. DOI: 10.1107/S2053229621005465/ep3017compound8sup9.hkl

Structure factors: contains datablock(s) compound9. DOI: 10.1107/S2053229621005465/ep3017compound9sup10.hkl

Structure factors: contains datablock(s) compound10. DOI: 10.1107/S2053229621005465/ep3017compound10sup11.hkl

Structure factors: contains datablock(s) compound11. DOI: 10.1107/S2053229621005465/ep3017compound11sup12.hkl

Supporting information file. DOI: 10.1107/S2053229621005465/ep3017sup13.pdf

CCDC references: 2085625, 2085624, 2085623, 2085622, 2085621, 2085620, 2085619, 2085618, 2085617, 2085616, 2085615

## Figures and Tables

**Table:** Experiments were carried out using a Rigaku XtalLAB Synergy-S diffractometer at 100 K, except for the data for compounds **1** and **2**, which were collected on a Rigaku Supernova diffractometer at 130 K. Cu *K*α radiation was employed, with the exception of the data collection for compound **8**, which used Mo *K*α radiation. H atoms were treated by a mixture of independent and constrained refinement.

	**1**	**2**	**3**	**4**
Crystal data				
Chemical formula	[Li_2_(C_7_H_4_O_3_)(H_2_O)_2_]	[Li_2_(C_7_H_4_O_3_)(H_2_O)_3_]	[Li(C_7_H_5_O_3_)]	[Na(H_2_O)_4_](C_7_H_5_O_3_)
*M*_r_	186.01	204.03	144.05	232.16
Crystal system, space group	Orthorhombic, *P* *b* *c* *a*	Orthorhombic, *P* *b* *c* *a*	Monoclinic, *P*2_1_/*c*	Triclinic, *P*\overline{1}
*a*, *b*, *c* (Å)	7.1897 (3), 11.7989 (5), 18.5349 (8)	13.9791 (6), 7.4348 (3), 18.2797 (7)	14.8904 (4), 5.0487 (1), 8.4721 (2)	6.7058 (2), 6.8114 (2), 12.5933 (3)
α, β, γ (°)	90, 90, 90	90, 90, 90	90, 99.281 (2), 90	83.361 (2), 75.966 (2), 72.945 (2)
*V* (Å^3^)	1572.33 (12)	1899.84 (13)	628.57 (3)	532.89 (3)
*Z*	8	8	4	2
μ (mm^−1^)	1.10	1.04	0.99	1.47
Crystal size (mm)	0.24 × 0.16 × 0.07	0.27 × 0.10 × 0.06	0.36 × 0.21 × 0.12	0.17 × 0.06 × 0.06

Data collection				
Absorption correction	Multi-scan (*CrysAlis PRO*; Rigaku OD, 2015 ▸)	Multi-scan (*CrysAlis PRO*; Rigaku OD, 2018 ▸)	Analytical [*CrysAlis PRO* (Rigaku OD, 2018 ▸), based on expressions derived by Clark & Reid (1995 ▸)]	Multi-scan (*CrysAlis PRO*; Rigaku OD, 2018 ▸)
*T*_min_, *T*_max_	0.905, 1.000	0.869, 1.000	0.760, 0.924	0.880, 1.000
No. of measured, independent and observed [*I* > 2σ(*I*)] reflections	3789, 1588, 1412	4091, 1874, 1672	4598, 1321, 1235	5395, 2117, 1704
*R*_int_	0.019	0.019	0.027	0.058
(sin θ/λ)_max_ (Å^−1^)	0.629	0.625	0.634	0.634

Refinement				
*R*[*F* ^2^ > 2σ(*F* ^2^)], *wR*(*F* ^2^), *S*	0.029, 0.084, 1.04	0.031, 0.084, 1.04	0.033, 0.093, 1.07	0.042, 0.116, 1.06
No. of reflections	1588	1874	1321	2117
No. of parameters	143	160	104	175
No. of restraints	6	7	1	13
Δρ_max_, Δρ_min_ (e Å^−3^)	0.23, −0.29	0.30, −0.21	0.22, −0.27	0.25, −0.33

**Table:** 

	**5**	**6**	**7**	**8**
Crystal data				
Chemical formula	[K(C_7_H_5_O_3_)(H_2_O)_3_]	[K(C_7_H_5_O_3_)(H_2_O)]	[Rb(C_7_H_5_O_3_)(H_2_O)]	[Cs(C_7_H_5_O_3_)(H_2_O)]
*M*_r_	230.26	194.23	240.60	288.04
Crystal system, space group	Monoclinic, *P*2_1_/*c*	Orthorhombic, *P* *b* *c* *a*	Monoclinic, *P*2_1_/*c*	Monoclinic, *P*2_1_/*c*
*a*, *b*, *c* (Å)	12.34808 (14), 11.25501 (14), 7.07200 (8)	10.0126 (2), 7.6695 (2), 20.0996 (4)	10.1069 (1), 10.0060 (1), 8.0198 (1)	10.1271 (2), 10.1220 (2), 8.6270 (1)
α, β, γ (°)	90, 102.1980 (11), 90	90, 90, 90	90, 98.557 (1), 90	90, 102.953 (2), 90
*V* (Å^3^)	960.66 (2)	1543.48 (6)	802.01 (2)	861.82 (3)
*Z*	4	8	4	4
μ (mm^−1^)	4.94	5.83	8.30	4.27
Crystal size (mm)	0.31 × 0.24 × 0.08	0.33 × 0.21 × 0.04	0.47 × 0.15 × 0.05	0.34 × 0.23 × 0.13

Data collection				
Absorption correction	Multi-scan (*CrysAlis PRO*; Rigaku OD, 2021 ▸)	Multi-scan (*CrysAlis PRO*; Rigaku OD, 2018 ▸)	Gaussian (*CrysAlis PRO*; Rigaku OD, 2018 ▸)	Multi-scan (*CrysAlis PRO*; Rigaku OD, 2018 ▸)
*T*_min_, *T*_max_	0.509, 1.000	0.444, 1.000	0.121, 1.000	0.666, 1.000
No. of measured, independent and observed [*I* > 2σ(*I*)] reflections	6174, 1952, 1878	6496, 1615, 1439	5073, 1615, 1543	22548, 5490, 4758
*R*_int_	0.029	0.072	0.031	0.044
(sin θ/λ)_max_ (Å^−1^)	0.634	0.634	0.634	0.921

Refinement				
*R*[*F* ^2^ > 2σ(*F* ^2^)], *wR*(*F* ^2^), *S*	0.031, 0.085, 1.05	0.052, 0.147, 1.07	0.025, 0.070, 1.10	0.027, 0.065, 1.05
No. of reflections	1952	1615	1615	5490
No. of parameters	155	121	121	121
No. of restraints	7	4	4	4
Δρ_max_, Δρ_min_ (e Å^−3^)	0.46, −0.41	0.61, −0.57	0.42, −0.73	0.81, −1.58

**Table:** 

	**9**	**10**	**11**
Crystal data			
Chemical formula	[Na(C_7_H_5_O_3_)(C_7_H_6_O_3_)(H_2_O)_2_]·H_2_O	[K(C_7_H_5_O_3_)(C_7_H_6_O_3_)]·H_2_O	[Rb(C_7_H_5_O_3_)(C_7_H_6_O_3_)]·H_2_O
*M*_r_	352.26	332.34	378.71
Crystal system, space group	Monoclinic, *P*2_1_/*n*	Monoclinic, *P*2/*c*	Monoclinic, *P*2/*c*
*a*, *b*, *c* (Å)	7.6704 (2), 10.1413 (3), 19.8263 (4)	16.4136 (4), 3.76614 (9), 11.1651 (3)	16.3445 (5), 3.8267 (1), 11.3460 (3)
α, β, γ (°)	90, 92.001 (2), 90	90, 92.533 (2), 90	90, 94.437 (2), 90
*V* (Å^3^)	1541.31 (8)	689.51 (3)	707.51 (3)
*Z*	4	2	2
μ (mm^−1^)	1.34	3.71	5.14
Crystal size (mm)	0.15 × 0.08 × 0.03	0.51 × 0.07 × 0.03	0.51 × 0.05 × 0.04

Data collection			
Absorption correction	Multi-scan (*CrysAlis PRO*; Rigaku OD, 2018 ▸)	Gaussian (*CrysAlis PRO*; Rigaku OD, 2021 ▸)	Analytical [*CrysAlis PRO* (Rigaku OD, 2018 ▸), based on expressions derived by Clark & Reid (1995 ▸)]
*T*_min_, *T*_max_	0.476, 1.000	0.453, 1.000	0.366, 0.830
No. of measured, independent and observed [*I* > 2σ(*I*)] reflections	10034, 3135, 2564	3764, 1399, 1311	6047, 1456, 1389
*R*_int_	0.068	0.033	0.055
(sin θ/λ)_max_ (Å^−1^)	0.633	0.634	0.632

Refinement			
*R*[*F* ^2^ > 2σ(*F* ^2^)], *wR*(*F* ^2^), *S*	0.055, 0.141, 1.05	0.039, 0.111, 1.06	0.036, 0.097, 1.11
No. of reflections	3135	1399	1456
No. of parameters	253	114	110
No. of restraints	11	2	2
Δρ_max_, Δρ_min_ (e Å^−3^)	0.36, −0.38	0.45, −0.36	0.50, −0.69

Computer programs: *CrysAlis PRO* (Rigaku OD, 2015 ▸, 2018 ▸, 2021 ▸), *SHELXT2014* (Sheldrick, 2015*a* ▸), *SHELXL2018* (Sheldrick, 2015*b* ▸), *OLEX2* (Dolomanov *et al.*, 2009 ▸) and *CrystalMaker* (Palmer, 2020 ▸).
