# Supplementary material for: Alkali metal salts of 4-hy­droxy­benzoic acid: a structural and educational study
Source: Acta Crystallogr C Struct Chem. 2021 Jun 9;77(Pt 7):340–53. doi: 10.1107/S2053229621005465 (PMC8254528; doi:10.1107/S2053229621005465)
Supplement: Supplementary file 13 [file c-77-00340-sup13.pdf]

Acta Cryst C

STRUCTURAL CHEMISTRY

Volume 77 (2021)

Supporting information for article:

Alkali metal salts of 4-hydroxybenzoic acid: a structural and educational study

Brendan F. Abrahams, Christopher J. Commons, Timothy A. Hudson, Robin Sanchez Arlt, Keith F. White, Michael Chang, John J. Jackowski, Matthew Lee, Shang X. (Matthew) Lee, Harrison D. Liu, Bill M. Mei, Joshua E. Meng, Lincoln Poon, Xiaolin (Jerry) Xu and Zekai (Kenny) Yu

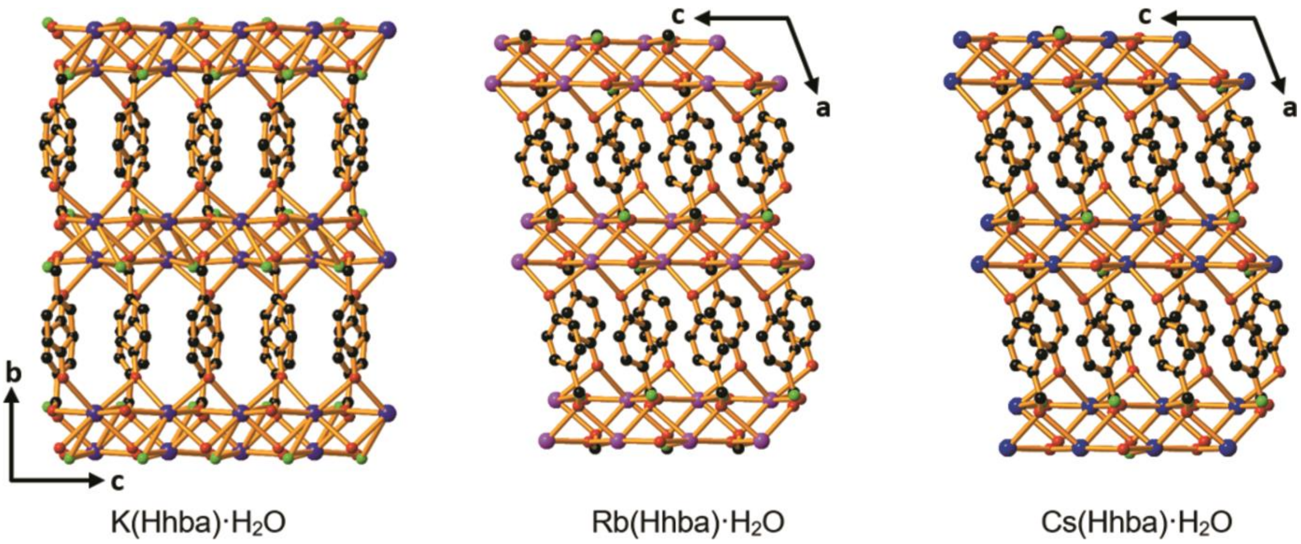

Figure S1

A comparison of the structures of compounds 6, K(Hhba)(H_2O), 7, Rb(Hhba)(H_2O), and 8, Cs(Hhba)(H_2O); note that compounds 7 and 8 are isostructural. Each layer is approximately 10.0 Å apart. Colour code: K purple, Rb pink, Cs blue, carboxylate and phenolate O red, water O green, C black. Hydrogen atoms have been omitted.
